# Editorial: The utilization of plants in vaccine research

**DOI:** 10.3389/fpls.2023.1335247

**Published:** 2023-11-23

**Authors:** Sezer Okay, Aslıhan Kurt-Kızıldoğan, Bernardo Bañuelos-Hernández, Linda Avesani

**Affiliations:** ^1^ Department of Vaccine Technology, Vaccine Institute, Hacettepe University, Ankara, Türkiye; ^2^ Enzyme and Microbial Biotechnology Section, Department of Agricultural Biotechnology, Faculty of Agriculture, Ondokuz Mayıs University, Samsun, Türkiye; ^3^ Laboratory of Molecular Biology, Veterinary Faculty, La Salle Bajío University, León Gto, Mexico; ^4^ Department of Biotechnology, University of Verona, Verona, Italy

**Keywords:** plant-based vaccines, virus-like particles, VLPs, antigens, vaccine development, edible vaccines, mucosal immunity, oral immunization

In the framework of active immunization, plants provide a valuable platform that can serve as bio-factory for producing virus-like particles (VLPs) or subunit vaccines and they can also facilitate the direct delivery of these molecules through oral administration.

Leveraging plants in vaccine development presents numerous benefits, as they enable the cost-effective production of substantial quantities of target antigens. Additionally, the robust cell walls of these hosts provide protection against proteases in the gastrointestinal tract, rendering plants advantageous for implementing oral vaccination strategies.

Plant-derived antigens are safer compared to methods using mammalian cells/tissues which may contain viruses or prions. Additionally, genetic engineering of plants is relatively easy, and they possess the machinery for the post-translational modification of proteins.

In this scenario, our Research Topic aims to gather all current research efforts aimed to enhance the utilization of plant biotechnology in active immunization approaches.

The current Research Topic includes two reviews, one mini review, one clinical trial, and four original research studies focusing on plant-made vaccines intended either for human or animal use and on strategies in which plants may be used to optimize antigens delivery.

In the four original research articles present in our Research Topic and in the Clinical Trial report, some practical examples of the potential of plant molecular pharming as a robust system in vaccine research is provided.

In the Clinical Trial report, Leroux-Roels et al. report the results of a phase I, double-blind, placebo-controlled clinical trial, performed to test the Safety and immunogenicity of a bivalent, plant-produced VLP, NoV vaccine at two dose levels without adjuvant. The data show that a single dose of the vaccine is sufficient to reach a peak immune response thus paving the way to further evaluation of the non-adjuvanted vaccine candidate. This study illustrates that the utilization of plant-based vaccines is advancing towards clinical use, with the potential to soon contribute to global vaccination initiatives.

Pazos-Castro et al. published an original research article in which the ‘Pru p 3’ allergen, displayed by Turnip mosaic virus-derived VLPs was produced in *Nicotiana benthamiana*, and the purified TuMVc -Pru p 3 VLPs could effectively reduce allergic responses in mice, showing notable immunomodulatory properties.

Kaldis et al. describe expression of a recombinant subunit vaccine composed of *Mannheimia haemolytica* leukotoxin A (LktA) and lipoprotein PlpE fragments fused to a cholera toxin mucosal adjuvant (CTB) within plant tissue to induce robust humoral and mucosal immune responses when administered to mice. These promising results justify further investigation and potential application in cattle.

By keeping in mind the prospective benefits of using plants as a platform for vaccine production, VanderBurgt et al. cover strategies for expressing and purifying plant-made vaccines, encompassing transgenic, transient, and cell suspension culture methods, targeting different plant tissues like leaves and seeds, ultimately highlighting the potential future advantages of plant-based vaccine production.

In the framework of process development, Schwestka et al. explore the potential of plants to generate protein storage organelles like protein bodies (PBs) for encapsulating recombinant proteins. Their paper outlines how they have combined various PB-forming proteins in *N. benthamiana* to advance this technology, resulting in the development of different multi-layered protein structures. Additionally, they demonstrate the incorporation of vaccine antigens into these multi-layered PBs. This method holds promise for the creation of tailored PBs that can serve as drug delivery systems, accommodating the integration of multiple functional elements.

The reviews and mini-review provide a comprehensive and evaluative perspective on the particular applications of plant-based vaccination for both animals and humans, as well as aspects related to the production process.

Su et al. make a compilation of the different viral antigens that have been produced in plants to treat human and animal diseases, highlighting the production of vaccines against the recent SARS-CoV-2 pandemic and the hepatitis B and C virus (HBV and HCV), of which progress has been made to the clinical study of vaccines. In the veterinary area, the generation of low-cost vaccines is of great importance and this review addresses the work carried out for the development of vaccines for the prevention of diseases with important implications in zoonosis as well as economic losses.

Venkataraman et al. in their review, address the main platforms that use plant cells for protein production. Antigens can be produced in the entire plant (leaves), in the seeds or in plant cells in culture. Until now, transient expression based on viral vectors and the *N. benthamiana* plant has been the most successful and used by biotechnology companies for the production of vaccines that are in the clinical phase. Another aspect considered is the purification processes of post-production antigens in the plant. The aspects of purification, clarification, sedimentation, filtration and flocculation are considered.

Bolaños-Martínez and Strasser published a mini-review article providing insights into recent efforts to develop plant-made poliovirus candidates, with an emphasis on strategies to optimize the production of viral antigens.

Overall, the Research Topic gives an updated and comprehensive overview of the current perspectives and achievements of plant molecular farming underling the challenges and the opportunities for achieving its full potential for human and animal health.

## Author contributions

SO: Writing – original draft. AK-K: Writing – original draft. BB-H: Writing – original draft. LA: Writing – original draft.

